# Root system size response of *bzh* semi-dwarf oilseed rape hybrids to different nitrogen levels in the field

**DOI:** 10.1093/aob/mcy197

**Published:** 2018-11-17

**Authors:** Antje Schierholt, Tina Tietz, Gerd Patrick Bienert, Andreas Gertz, Sebastian Miersch, Heiko C Becker

**Affiliations:** 1 Georg-August Universität Göttingen, Department of Crop Sciences, Göttingen, Germany; 2 Metalloid Transport Group, Department of Physiology and Cell Biology, Leibniz Institute of Plant Genetics and Crop Plant Research, Gatersleben, Germany; 3 KWS SAAT SE, Einbeck, Germany

**Keywords:** *Brassica napus*, electrical capacitance, nitrogen efficiency, nitrogen uptake efficiency, canola, dwarfing genes, root system, growth type

## Abstract

**Background and Aims:**

In oilseed rape (*Brassica napus*) semi-dwarf hybrid varieties from crosses between *bzh* dwarf and normal-type lines are of increasing interest. They have improved nitrogen (N) uptake, N-utilization and N-use efficiency compared to normal types. This study aimed to elucidate whether these N-related effects can be explained by the *bzh* shoot growth-type alone or also by differences in root traits.

**Methods:**

Root system size was measured using root electrical capacitance (EC) in field trials with two N levels in two sets of genotypes segregating for the *bzh*-locus: (1) 108 doubled haploid (DH) test hybrids in two seasons, 2010–2012, and (2) 16 near-isogenic hybrids in the 2016–17 season. Quantitative trait loci (QTL) for root EC were estimated in DH test hybrids. Seedling root architecture parameters were monitored *in vitro*.

**Key Results:**

*In vitro* root growth showed a higher root: shoot ratio in *bzh* semi-dwarf hybrids. Root EC in field trials was higher at high N supply than at zero N fertilization. In most trials semi-dwarf hybrids had higher EC than normal-type hybrids, but they reduced root EC in response to N limitation more than normal types. Root EC was more heritable at the end of flowering (*h*^2^ = 0.73) than at the beginning of flowering (*h*^2^ = 0.36) in near-isogenic hybrids and had a lower heritability in trials of DH test hybrids (*h*^2^ = 0.27). A QTL for root EC in the genomic region of the *bzh*-locus on linkage group A06 was significant at zero N fertilization.

**Conclusions:**

Root EC proved to be a meaningful method in oilseed rape breeding programmes targeting root system size. The greater reduction of semi-dwarf root EC compared to the normal type under low N supply with simultaneous increase in N efficiency implies that in roots it is not a question of ‘the more the merrier’ and that the *bzh* root system reacts highly economically when N is scarce.

## INTRODUCTION

In Brassicaceae, many different dwarfing genes have been described, but only the *bzh* gene is used in released oilseed rape hybrid varieties in Europe (Foisset *et al.*, 1995; Miersch *et al.*, 2016*a*). Oilseed rape (*Brassica napus*) *bzh* semi-dwarf hybrids have improved winter-hardiness and drought tolerance (Pinochet and Renard, 2012), and they show equal yield, higher harvest index and higher nitrogen (N) use efficiency compared to normal-type hybrids (Miersch *et al*., 2016*a*, *b*).

Dwarfing genes have been well known in crops such as wheat (*Triticum aestivum*), rice (*Oryza sativa*) and barley (*Hordeum vulgare*) since the green revolution (Hedden, 2003). Their use in plant breeding resulted in shorter and more lodging-resistant genotypes that could be fertilized intensively with N to gain higher yields. In wheat, most varieties carry the *Rht* (reduced height) gene homozygously in either the B or the D genome (Rht-B1 or Rht-D1; Peng *et al.*, 1999; Wojciechowski *et al.*, 2009). *Rht* is a repressor of the gibberellic acid signalling pathway (*rga*) gene homologue, as is the *bzh* gene in oilseed rape (Renard *et al.*, 2010). These genes encode transcription factors belonging to the DELLA subgroup of the GRAS superfamily (Peng *et al.*, 1999; GAI-RGA-SCARECROW), known as negative regulators in the gibberellic acid response. In contrast to the homozygous state of *Rht* in wheat, *bzh* in oilseed rape is heterozygous in semi-dwarf hybrid varieties, because the homozygous *bzh* dwarf type is extremely short and very low yielding. The higher N use efficiency of *bzh* semi-dwarfs could thus be explained by the reduced shoot matter, which costs the plant less N and assimilates. Alternatively, an altered root morphology induced by the *bzh* locus could explain the higher N use efficiency. In wheat, Wojciechowski *et al.* (2009) analysed early plant shoot and root growth of near-isogenic lines encoding *Rht-B1b*, *Rht-B1c*, *Rht-D1b* and *Rht-D1c* in soil columns from the glasshouse and field, and found an increase in root dry matter resulting in a significant increase in the root: total dry matter ratio in *Rht-B1b*, *Rht-B1c* and *Rht-D1c*, compared to the *rht* control. Moreover, the total root length of *Rht-B1c* and *Rht-D1c* was significantly shorter in soil columns and field trials, indicating a denser rooting system in the *Rht* dwarf types. In barley, a greater number of ‘green revolution’ dwarfing genes are known (Xu *et al.*, 2017). Chloupek *et al.* (2006) analysed root system size by use of root electrical capacitance (EC) in a barley doubled haploid (DH) population segregating for the shoot dwarf locus *sdw1* and found a quantitative trait locus (QTL) for root system size that co-segregated with the shoot dwarf gene and increased root system size in early growth stages. Barley dwarf locus *sdw1* encodes a gibberellin oxidase (Xu *et al.*, 2017). In oilseed rape, a comparison of one *bzh* semi-dwarf hybrid and two normal-type hybrid varieties by Hofmann and Christen (2007) indicated that the *bzh* gene increased the autumn root length growth of rapeseed in a late sown field experiment under unfavourable conditions in 2003 (semi-dwarf variety *Belcanto* had significantly longer roots than two normal type varieties), whereas there were no significant effects under favourable conditions (early sowing). Dresbøll *et al.* (2015) analysed root growth in minirhizotrons to 2.3-m soil depth in a field study with two N levels and found no significant difference in root growth between one *bzh* semi-dwarf and two normal-type hybrids throughout the season. In addition to genetic factors, root growth and architecture are also affected by the environment, such as by the N fertilization level (Gruber *et al.*, 2013). High N supply suppresses root branching, whereas during N limitation, the development of lateral roots is strongly enhanced. Root axis number, rooting depths, rooting density and root longevity are affected by changes in N availability (Miller and Cramer, 2004; Hawkesford *et al.*, 2012; Gruber *et al.*, 2013).

As roots grow hidden in the soil, they are very difficult to characterize in the field. Root biomass and root distribution in the soil profile can be estimated destructively or non-destructively in various artificial environments ranging from *in vitro* gel plates to rhizotrons, as reviewed by Fiorani and Schurr (2013). However, the more precise techniques are very laborious and expensive and therefore are not applied in plant breeding, where non-destructive methods for high numbers of genotypes in the field are required. Chloupek (1972) suggested the non-destructive measurement of root EC as an indirect measure for root system size (Chloupek *et al.*, 2006). According to Dalton’s (1995) physical model, root EC can be related to root biomass by defining each root as a cylindrical capacitor. Root EC is proportional to total root surface area, which is the sum of main, lateral and fine roots. Because electrical polarization of the root membranes depends on the geometric and dielectric proportions of the root system, root EC differs within and between crop species or developmental stages (Dalton, 1995; Postic and Doussan, 2016). Soil composition and soil humidity affect root EC significantly (Chloupek, 1972; Dalton, 1995; Dietrich *et al.*, 2013; Postic and Doussan, 2016); therefore, a high soil humidity and consistent soil composition within a trial are prerequisites for application of methods based on EC.

Root EC provides a cheap, non-destructive and relatively fast root phenotyping method to estimate root system size. It has been successfully applied in trees (Ellis *et al.*, 2013), herbaceous plants (Aulen and Shipley, 2012), and cereals such as barley (Chloupek *et al.*, 2006) and wheat (Postic and Doussan, 2016). The root type of *B. napus* differs from grasses, herbaceous plants and trees. Winter oilseed rape develops a tap root and a fine root system in autumn. In cold winters, the fine root system is reduced but it regrows in spring. Wu *et al.* (2016) measured root EC in oilseed rape in a glasshouse pot experiment and found high and significant linear correlations with root parameters, such as root biomass and fine root biomass. Rudloff (2016) found root EC as highly heritable under field conditions in an oilseed rape diversity set (*h*^2^ = 0.81 and 0.65 at the end of flowering and maturity, respectively). However, Chloupek (1972) and Rudloff (2016) suspected that root EC might be strongly influenced by the size of the tap root, and Wu *et al.* (2016) found a high correlation between the cross-sectional area at the basal stem and root EC, which raises the question of whether the oilseed tap root architecture makes the application of root EC less meaningful than in cereals (Postic and Doussan, 2016) or herbaceous plants (Aulen and Shipley, 2012).

The objectives of this study were as follows. (1) To analyse the effect of the *bzh* gene on roots and their responses to different N levels, by measuring root EC under zero and high N fertilization in the field. Based on QTL analysis, we tested whether loci causing differences in root EC between semi-dwarf and normal-type DH test hybrids are located in the region of the *bzh* dwarf gene. (2) To test the effect of stem diameter as an estimate of tap root size on root EC. (3) To analyse if the *bzh* gene has an effect on root architecture traits even at an early stage of plant development in an *in vitro* seedling growth test.

For these analyses, we measured root system size by use of root EC and stem diameter in field trials, with two N levels and two sets of genotypes that segregated for the *bzh* locus: (1) 108 test hybrids derived from a DH population and segregating into 54 *bzh* semi-dwarf types and 54 normal types in the 2010–11 and 2011–12 seasons, and (2) 16 test hybrids consisting of eight near-isogenic pairs of *bzh* semi-dwarf hybrids and normal-type hybrids in the 2016–17 season.

## MATERIAL AND METHODS

### Plant material

#### (1) DH test hybrids

A DH population was derived from the cross ‘Alesi-*bzh* × H30’. ‘Alesi-*bzh*’ is a dwarf line derived from the canola quality German winter oilseed cultivar ‘Alesi’ backcrossed (BC4) with the *bzh* dwarf mutant (Foisset *et al.*, 1995). ‘H30’ is a normal-type resynthesized rapeseed line. The DH lines segregated into dwarf and normal types. All DH lines were crossed with a normal-type, highly homozygous and male-sterile tester line. The hybrids segregated into semi-dwarfs (*bzh*/*Bzh*) and normal types (*Bzh*/*Bzh*). For plant material details, see Miersch *et al.* (2016*a*).

#### (2) Near-isogenic hybrid pairs

Eight pairs of near-isogenic test hybrids were developed by crossing normal-type (*Bzh*/*Bzh*) lines first with a normal-type (*Bzh*/*Bzh*) line and second with the respective near-isogenic dwarf-type (*bzh*/*bzh*) line. The eight near-isogenic hybrid pairs were obtained from crosses of six mother and four father parental lines (Supplementary Data Table S1). The dwarf lines were developed by crossing the *bzh* introgression line with the normal-type line, followed by two backcrosses. Each near-isogenic pair therefore consisted of a semi-dwarf (*bzh*/*Bzh*) and a normal-type (*Bzh*/*Bzh*) test hybrid.

### Field trials

Field experiments of 108 DH test hybrids were carried out during two seasons (2010–11, 2011–12) and experiments of 16 near-isogenic hybrids were grown in 2016–17, all at Reinshof experimental station in Göttingen, central Germany, on alluvial meadow soils. In 2011–12, an additional trial with a reduced set of 12 DH test hybrids was performed in Einbeck, 50 km north of Göttingen on a brunic aerosol. Precipitation and temperature differed between seasons (Table 1). Genotypes were grown at two N levels (zero and high N fertilization) and were unreplicated in the 2010–11 season but with two replicates in 2011–12 and 2016–17. Within each replication, growth types were interlaced by alternately growing one strip of semi-dwarfs and one strip of normal types. Experiments were designed as split–split plots with N level as a main plot factor, growth type as a sub-plot factor, and genotype within growth type as a sub-sub-plot factor. Genotypes within growth types were arranged in a lattice design in the 2011–12 season and in a randomized block design in 2010–11 and 2016–17. The plots consisted of two rows (2.5 m in length, 30 cm between rows, 6 cm between plants) in the seasons from 2010 to 2012. In the 2016–17 season, plots were designed as one row (7.5 m length, 75 cm between rows, 8 cm between plants). At Einbeck (2011–12), root EC was measured in yield plots (Miersch *et al.*, 2016*a*). In all trials, plants were sown by single seed drill for uniform spacing.

**Table 1.**
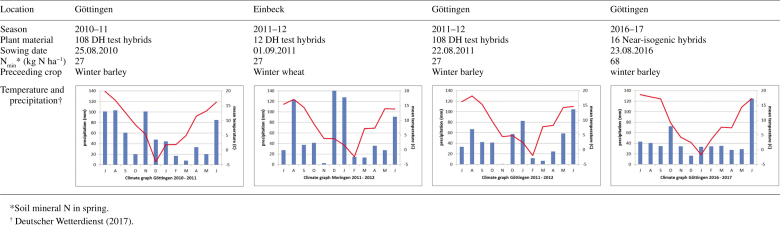
Overview of oilseed rape field trials at Göttingen and Einbeck at low and at high nitrogen (N) supply

Weed, disease and pest control, fertilization (with the exception of N), and all agronomic treatments for all trials followed the recommendations for winter oilseed rape production in Germany. The experiments were conducted with two levels of N fertilization: zero N and optimal N supply. At each location, the soil mineral N (N_min_) was determined in spring at the beginning of plant growth (Table 1). Soil samples were taken from a depth of 0–90 cm, and N_min_ was analysed following the method of VDLUFA (1991). At the low N level, no N fertilizer was applied. For the high N treatment, 177 kg N ha^−1^ was applied in all trials. About half of the N was applied as ammonium sulphate fertilizer plus calcium ammonium nitrate fertilizer in spring at the beginning of plant growth (BBCH 30; Lancashire *et al.*, 1991), and the other half was given at the beginning of shoot development (BBCH 32; calcium ammonium nitrate fertilizer). Biomass was determined as described in detail by Miersch (2015) and Miersch *et al.* (2016*b*), by separating seed and straw when harvesting at maturity.

### Root electrical capacitance and stem diameter

**Fig. 1.**
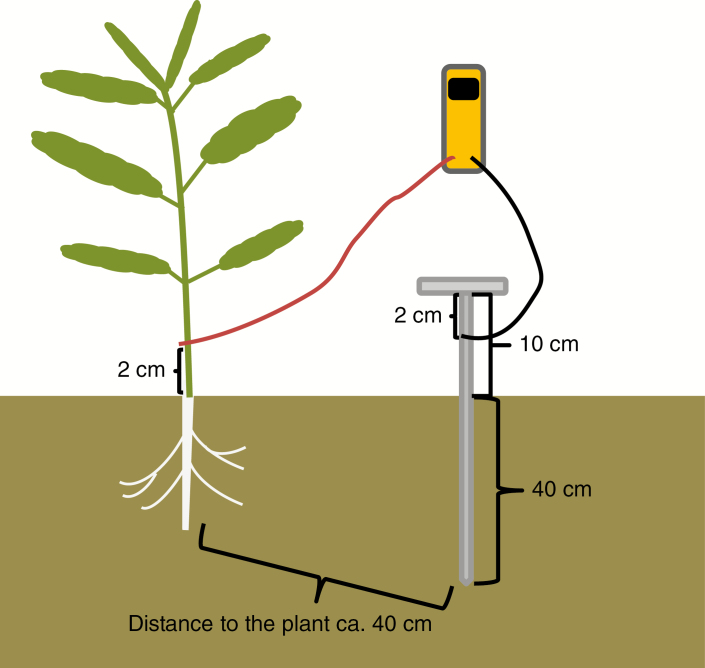
Root electrical capacitance measurements in the field. The plant electrode was attached with a clamp about 2 cm above ground at the hypocotyl of the rapeseed plant and the soil electrode was pushed approx. 40 cm into the ground.

Root EC was measured preferably after rainfall to provide uniform soil moisture. The method was applied as described by Rudloff (2016). Root EC was measured in the field during and after the flowering period (Table 2) using a hand-held capacitance meter (Dual Display LCR meter, ELC-132A; Escort instruments cooperation, Taipei, Taiwan) with a frequency of 1 kHz in alternating current. The plant electrode was fixed with a clamp about 2 cm above ground at the hypocotyl (Fig. 1), and the soil electrode was clamped to a steel stick that was pushed 40 cm into the soil at a distance of about 40 cm from the plant. Root EC was recorded in nano Farad (nF) when it became stable about 30 s to 1 min after attaching the two clamps. Root EC was measured in ten plants per plot in 2010–11 and in five plants in 2011–12; the mean value was used in the analysis of variance (ANOVA). In the trial of the near-isogenic hybrids in the 2016–17 season, root EC and stem diameter were recorded at the same time for 12 plants per row. Single plants were labelled, and plants in the same row were measured twice, at the beginning and at the end of flowering. Stem diameter was measured 2 cm above the ground at the hypocotyl.

**Table 2.** Mean root electrical capacitance and stem diameter and corresponding F-test results between growth types and N levels at zero nitrogen (N)–fertilization (N0) and at high N supply (N1)

Location	Date	Plant stage	Plant material	Growth type	Number of genotypes	Electrical capacitance (nF) and F-test for growth types		F-test for N levels	Stem diameter (cm) and F-test for growth types		F-test for N levels
						N0	N1		N0	N1	
Göttingen	27.06.2011	Beginning of maturity	DH test hybrids	Semi-dwarf	54	0.9	1.1	**			
		(BBCH 80)		Normal type	54	1.0*	1.2**	**			
Göttingen	22.05.2012	Full flowering	DH test hybrids	Semi-dwarf	54	2.3	3.5	*			
		(BBCH 65)		Normal type	54	2.5**	2.8**	^ns^			
Einbeck	03.07.2012	Beginning of maturity	DH test hybrids	Semi-dwarf	12	1.4	2.8	**			
		(BBCH 80)		Normal type	12	1.5**	2.2 ^ns^	^+^			
Göttingen	21.04.2017	Beginning of flowering	Near-isogenic hybrids	Semi-dwarf	8	3.4	6.8	**	1.4	1.9	^+^
		(BBCH 61)		Normal type	8	3.8 ^ns^	6.2*	**	1.8^+^	2.3^*^	*
Göttingen	06.06.2017	End of flowering	Near-isogenic hybrids	Semi-dwarf	8	4.0	5.3	^ns^	1.7	2.1	^+^
		(BBCH 69)		Normal type	8	4.1 ^ns^	4.9 ^ns^	^ns^	2.1^+^	2.4 ^ns^	^ns^

^+^Significant at the 0.1 probability level; *significant at the 0.05 probability level; **significant at the 0.01 probability level.

### *In vitro* root growth experiment

The experiment was conducted three times at the Leibniz Institute of Plant Genetics and Crop Plant Research Gatersleben, Germany. In each experiment, 16 genotypes (eight near-isogenic hybrid pairs) were grown with three replications of five seedlings per genotype. Due to missing plants, near-isogenic hybrid pair No. 1 was discarded from the analysis. Within each experiment, the spatial arrangement of the near-isogenic pairs was randomized.

**Fig. 2.**
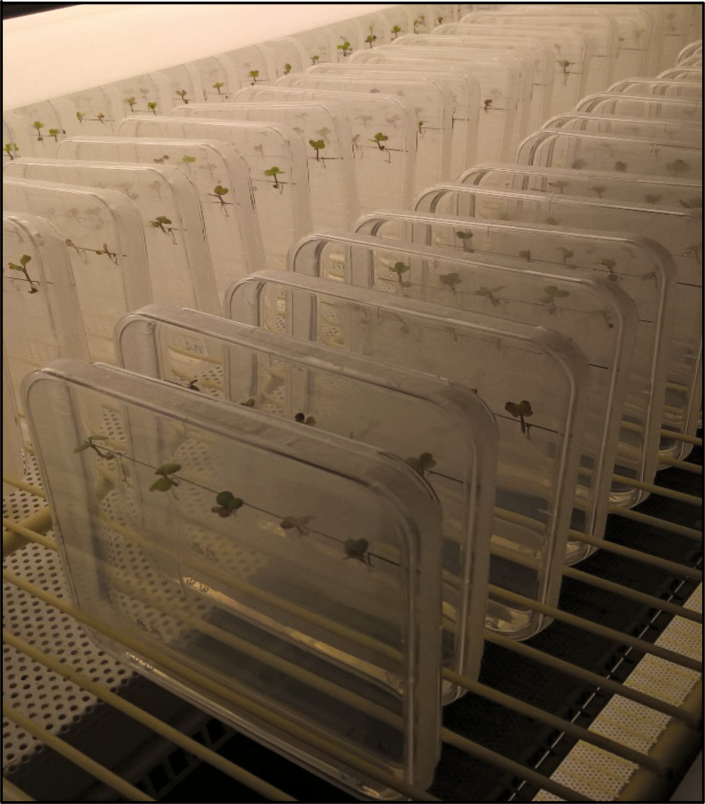
Root growth *in vitro* experiment. Five seedlings per genotype were grown in 12 × 12-cm Petri dishes under controlled conditions.

Sterilized seeds were pre-germinated in sterile plastic containers on a medium containing 50 % Hoagland solution and 0.3 % Agar-Agar (Carl Roth, Karlsruhe, Germany). Seeds were stratified without light at 4 °C. After 2 d, germinating seeds were moved to 15 °C and light for 15 h before seeds with identical root length (about 3–5 mm) were transferred into 12 × 12-cm Petri dishes filled with 50 mL medium, composed of 1/8 modified Hoagland solution, 0.6 % Agar-Agar, pH 5.8 [1× modified Hoagland solution contained MES (2 mm), Ca(NO_3_)_2_ (5 mm), MgSO_4_ (5 mm), KNO_3_ (5 mm), KH_2_PO_4_ (1 mm), FeNa-EDTA (100 µm), MnCl_2_ (4.5 µm), ZnSO_4_ (3.8 µm), H_3_BO_3_ (50 µm), CuSO_4_ (0.3 µm), (NH4)_6_Mo_7_O_24_ (0.1 µm)]. The layer of medium in the Petri dishes was sloped, and a stripe of 2 cm on the top side was without medium. On this edge of the medium, five seedlings (radicle between 2 and 5 mm) were positioned, and Petri dishes were sealed with Leukoplast tape. Petri dishes were scanned (Epson scanner, 600 dpi, black and white) for documentation of root tip length at the beginning of the experiment. Seedlings were vertically cultured at 15/12 °C day/night temperatures and a 10-h day length for 7 d (Fig. 2). The temperature in the three experiments was slightly different (2 °C lower in experiments 2 and 3 to slow down growth). After 7 d, cotyledons were discarded, and roots were carefully sorted so that single plants were separated and all roots were in one layer. Petri dishes were scanned (Epson scanner, 600 dpi, black and white). Image contrast was adjusted and marks were removed from the image using gimp software (gnu image manipulation software; https://www.gimp.org), thereby allowing a better quantification of roots. Root parameters were quantified using the EZ-Rhizo software (beta-Version 2; Armengaud *et al.*, 2009). Root tip length (cm), main root length (cm), number of lateral roots (>1 mm length), length of lateral roots (cm) and hypocotyl length (cm) were scored. Root tip length at the beginning of the experiment was subtracted from main root length to reduce the effects of variation in germination.

### Statistical analysis

Lattice calculations, ANOVA, Spearman’s rank correlation coefficients and estimates of heritability (*h*^2^) were calculated using PLABSTAT software (version 3A) (Utz, 2011). The effect of N level was tested in the trial of DH test hybrids using the following model for a slit-split plot design:

$${\text{Y}}_{\text{jklo}}=\text{µ}+{\text{n}}_{\text{o}}+{\text{b}}_{\text{jo}}+{\text{t}}_{\text{k}}+{\text{tn}}_{\text{ko}}+{\text{bt}}_{\text{jko}}+{\text{g}}_{\text{lo}}+{\text{gn}}_{\text{lko}}+{\text{gb}}_{\text{lkjo}}$$

in which Y_jklo_ is the observation of genotype l within growth type k in block j within N level o with the respective two- and three-fold interactions. Whole-plot error is denoted by b_jo_, sub-plot error is denoted by bt_jko_ and sub-sub-plot error is denoted by gb_lkjo_. The factors block and genotype were considered random.

The same model was applied in the set of near-isogenic hybrids with the following changes: because the same plants were always measured, single plant values were used in the ANOVA and the effect of plants was added to the sub-sub-plot error; the factor block was considered random.

### QTL analyses

QTL were estimated in the DH test hybrids. A linkage map was developed as described by Miersch *et al.* (2015) using 100 DH lines of the mapping population ‘Alesi-*bzh* × H30’. QTL detection was performed with WinQTL Cartographer software (version 2.5) (Wang *et al.*, 2006) by composite interval mapping with 1000 permutations, as described by Zeng (1994). The QTL mapping of test crosses was performed as established in hybrid crops such as maize (Schön *et al.*, 1994) or oilseed rape (Radoev *et al.*, 2008). QTL analyses were performed across environments and for single environments. The QTL confidence interval was defined as LOD-1 following Gadau *et al.* (2012). LOD thresholds were estimated for *P* = 0.05 and varied between LOD 2.5 (Göttingen 2012, zero N fertilization) and LOD 3.01 (and Göttingen 2011, zero N fertilization).

## RESULTS

### Variation in root electrical capacitance

Root EC as an indirect measure for root system size was greater at high N supply than at zero N fertilization, and the effect was significant in most tests, independent of location, plant material and growth type (Table 2). The highest relative differences between zero and high N supply were detected at the beginning of flowering, whereas the differences became smaller towards the end of the growing season/maturity. Absolute root EC values cannot be compared between trials and dates due to high environmental effects such as soil moisture, soil type or temperature. In all trials, N was by far the biggest source of variation (Table 3). The effect of genotype was smaller than the effect of growth type in DH test hybrids.

Thus, the direction of the effect of N on root EC was identical in all trials, independent of genotype (DH test hybrid or near-isogenic hybrid) and environment, i.e. root EC was increased by higher N fertilization. However, within N levels, the reaction of the two growth types differed and semi-dwarfs reduced root EC in response to N limitation more than normal types (Table 2). The only exception to this trend was found in Göttingen in the 2010–11 season, where normal-type DH test hybrids had a higher root EC than semi-dwarf hybrids at both N levels. The mean relative differences in root EC between growth types were higher at high N fertilization than at low N supply (Table 2).

The heritability of root EC was higher in the trials of near-isogenic hybrids (*h*^2^ = 0.36 and 0.73 at the beginning and at the end of flowering, respectively; Table 3) than in DH test hybrids (*h*^2^ = 0.27). This is mainly due to the larger genetic variance in near-isogenic hybrids, especially at the end of flowering (Table 3). Moreover, in the near-isogenic hybrids 12 plants per plot were measured compared to six plants in the DH test hybrids, which reduced the experimental error.

**Table 3.** Components of variance (Var.cp.) and respective *F* test results of root electrical capacitance from the analysis of variance combined for low and high nitrogen supply

Source	DH test hybrids							Near-isogenic hybrids		
	Göttingen 2010–11		Göttingen 2011–12		Einbeck 2011–12			Göttingen 2016–17		
	d.f.	Var.cp	d.f.	Var.cp	d.f.	Var.cp		d.f.	BBCH 61 Var.cp	BBCH 69 Var.cp
**Across nitrogen levels**										
Nitrogen (N)	1	0.021^+^	1	0.323*	1	0.545**	Nitrogen (N)	1	4.240**	0.293
Growth type (T)	1	0.006**	1	0.032**	1	0.025^+^	Growth type (T)	1	0	0
Genotype (G):T	53	0	106	0.013*	22	0.011	Genotype (G)	7	0.052**	0.125**
T × N	1	0	1	0.185**	1	0.109*	T × N	1	0.215*	0
N × G: T	104	0.056	106	0	22	0	G × N	7	0	0.045*
							G × T	7	0.019	0.155**
*h* ^*2*^_*G:T*_		–		0.27		0.27	*h* ^*2*^_*G*_		0.36	0.73
**Low nitrogen supply**										
T	1	0.0036*	1	0.0150**	1	0.008**	T	1	0.051	0
G:T	52	0.0514	53	0.0193+	23	0	G	7	0	0.035
							G × T	7	0	0.180*
*h* ^*2*^_*G:T*_		–		0.26		–	*h* ^*2*^_*G*_		0.02	0.30
**High nitrogen supply**										
T	1	0.0076**	1	0.233**	1	0.162	T	1	0.134*	0
G:T	52	0.0487	53	0.0156	23	0	G	7	0.051	0.228**
							G × T	7	0.032	0.129*
*h* ^*2*^_*G:T*_		–		0.15		–	*h* ^*2*^_*G*_		0.50	0.78

In total, 108 DH test hybrids (54 semi-dwarf and 54 normal type) and 16 near-isogenic hybrids (eight semi-dwarf and eight normal type) were tested. Negative estimates for variance components are given by ‘0’. Heritabilities were estimated within growth-type in DH test hybrids (*h*^2^_G:T_) and for 16 near-isogenic hybrids (*h*^2^_G_). The complete ANOVA across N levels is presented in Supplementary Data Table S2.

^+^Significant at the 0.1 probability level; *significant at the 0.05 probability level; **significant at the 0.01 probability level.

### Correlation between root EC and stem diameter

Stem diameter was measured only in near-isogenic hybrids, where it proved to be significantly higher at high N supply than at zero N fertilization (Table 2). The difference between N levels was higher at the beginning of flowering than at the end of flowering. Stem diameter increased highly significantly (*P* = 0.01) from the beginning to the end of flowering. This developmental-dependent gain in stem diameter was higher at zero N fertilization than with sufficient N supply, where just a small significant increase was observed (Table 2). The mean stem diameter of semi-dwarf genotypes was smaller than in normal-type near-isogenic hybrids on both dates and at both N levels.

**Fig. 3.**
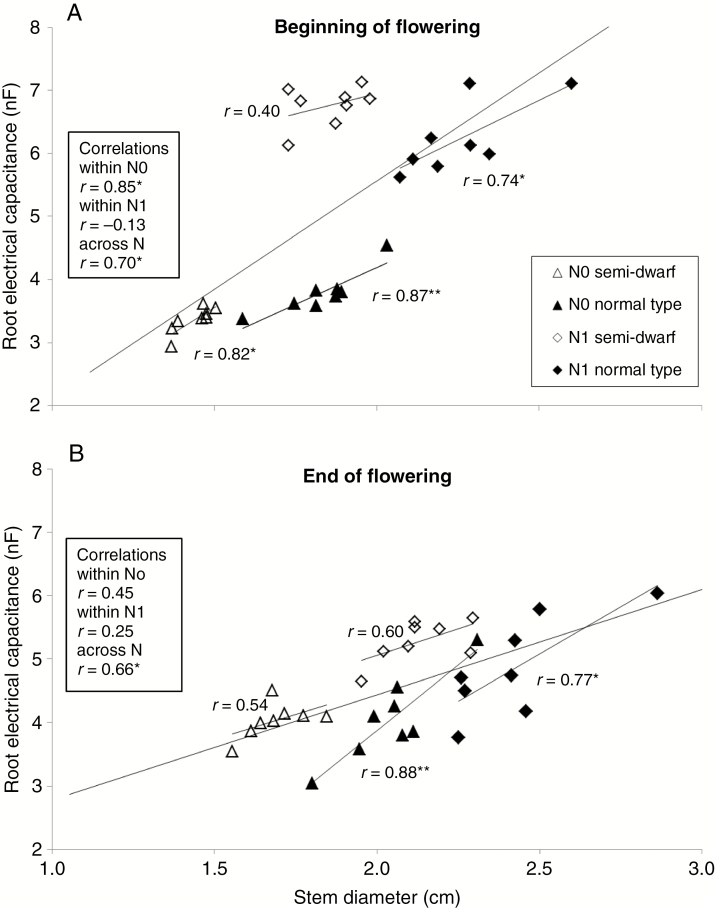
Root electrical capacitance and stem diameter correlations and regression lines of eight near-isogenic pairs of semi-dwarf and normal-type hybrids tested in 2016–17 at Göttingen at zero N-fertilization (N0) and at high N supply (N1). Measurements were conducted (A) at the beginning of flowering (BBCH 61) and (B) at end of flowering (BBCH 69). Lines are regression lines.

The overall correlation between stem diameter and root EC was slightly higher at the beginning compared to the end of flowering (*r* = 0.70 and 0.66, respectively; Fig. 3). Within the sub-groups of growth type and N level, correlation coefficients varied from *r* = 0.40 (semi-dwarf hybrids, zero N fertilization, beginning of flowering) to *r* = 0.88 (normal type, zero N fertilization, end of flowering) with no obvious trend. Across growth types at high N fertilization, the correlations were small (*r* = −0.13 and 0.25 at the end of flowering and at maturity, respectively).

### QTL for root electrical capacitance in DH test hybrids

QTL for root EC were estimated across growth types for two N levels and in two environments. At zero N fertilization, significant QTL across environments were only detected within the genomic region of the *bzh*-locus on linkage group A06 (maximum LOD 3.1), which explained 14 % of the phenotypic variation (Table 4). Across environments, the normal-type parent had an increased root EC. Within single environments, the QTL at A06 were non-significant, but additional QTL at linkage groups A03 and C08 were detected, which explained 15 and 17 % of the phenotypic variation, respectively (Table 4). At high N fertilization, a significant QTL was detected within the genomic region of the *bz*h-locus in one environment, i.e. Göttingen 2011–12, which explained 27 % of the phenotypic variation of root EC. Here, the allele of the dwarf-type parent had an increasing effect on root EC. In 2010–11 and across environments, QTL were detected within the genomic region of the *bzh*-locus but were not significant.

**Table 4.** *bzh*-locus and QTL for root electrical capacitance (EC) estimated for 100 DH test hybrids

QTL estimation environment	Linkage group	Confidence interval	Position (cM)	maximum LOD+	Effect*	*R* ^2^^**^
*bzh*-locus	A06		3.0			
**Low nitrogen supply**						
Göttingen 2010–11	A03	1–8	5	3.7	0.083	0.15
*Göttingen 2010–11*	*A06*	*–*	*3.1*	*1.5*	*0.027*	*0.07*
*Göttingen 2011–12*	*A06*	*–*	*2.4*	*2.3*	*0.059*	*0.10*
Göttingen 2011–12	C08	15–34	26	3.2	−0.077	0.17
Mean N0	A06	0–11	3.0	3.1	0.026	0.14
**High nitrogen supply**						
*Göttingen 2010–11*	*A06*	*–*	*3.3*	*1.0*	*0.051*	*0.05*
Göttingen 2011–12	A06	0–27	3.0	6.6	−0.169	0.27
Mean N1	A06	–	3.0	2.0	−0.057	0.09

Hybrids were grown at low nitrogen (N0) and high nitrogen (N1) supply at Göttingen in 2010–11 and 2011–12. QTL are given for single environments and as mean across environments. Non-significant QTL are in italic type.

^+^ Significant at the 0.05 probability level.

^*^ Effect of substituting the allele of dwarf type parent ‘Alesi-*bzh*’ by the allele of normal type parent ‘H30’.

^**^ Proportion of phenotypic variance explained by the QTL.

### *In vitro* seedling root growth

A comparison of seven near-isogenic pairs of hybrids revealed significantly longer main and lateral roots and a higher number of lateral roots in normal-type hybrids than in semi-dwarfs (Table 5). Differences between growth types after 7 d of growth on Hoagland medium were significant (*P* = 0.1) but small (<1 mm), and all traits were highly heritable (*h*^2^ = 0.74–0.94; Supplementary Data Table S3). The hypocotyl was highly significantly (*P* = 0.01; Table 5) shorter in semi-dwarf genotypes (21 % reduction). A smaller growth reduction effect of the *bzh* gene on root growth was monitored in the seedlings (3 % in main root length and 6 % in total root length), which resulted in a significantly higher root: shoot ratio in *bzh* semi-dwarfs than in normal-type near-isogenic hybrids. The axial position of lateral roots for estimation of a stunting effect on root growth could not be estimated in this experiment.

**Table 5.** Mean values and F-test results of seedling root parameters of seven *in vitro* grown near-isogenic pairs of *Brassica napus* test hybrids; the ANOVA is presented in Supplementary Data Table S3

Growth- type	Main root length (cm)	Sum of lateral root length (cm)	Total root length (cm)	Number of lateral roots	Hypocotyle length (cm)	Root: shoot ratio
Semi-dwarf	7.39	3.68	11.07	8.91	0.68	16.7
Normal type	7.60+	4.16+	11.75*	10.44*	0.86**	14.1**

^+^Significant at the 0.1 probability level; *significant at the 0.05 probability level; **significant at the 0.01 probability level.

### Correlation between root electrical capacitance and N efficiency parameters

**Fig. 4.**
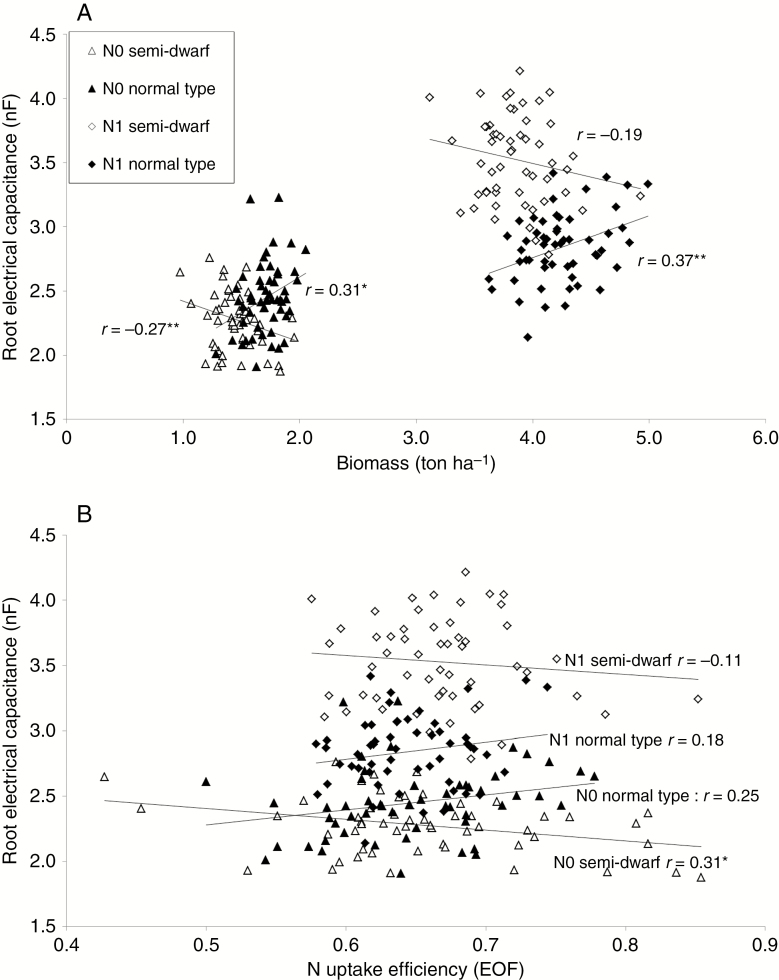
Correlations of root electrical capacitance and (A) biomass yield (Mg ha^−1^) and (B) nitrogen uptake efficiency at the end of flowering (EOF). In total, 54 semi-dwarf and 54 normal-type hybrids were tested at low (N0) and high N supply (N1) in Göttingen (2011–12). N efficiency data from Miersch (2014). Lines are regression lines.

In Göttingen in 2011–12, biomass was harvested at the end of flowering and at maturity in 108 DH test hybrids (Miersch, 2015; Miersch *et al*., 2016*b*; Supplementary Data Table S4) and N uptake efficiency at the end of flowering was correlated with root EC. Within normal-type DH test hybrids, root EC and shoot biomass were correlated positively at low and at high N supply. The direction of the correlation was inverse in semi-dwarfs, where root EC was reduced when shoot biomass was increased (Fig. 4). No significant correlation was detected between root EC (end of flowering measurement) and N efficiency parameters at maturity (Supplementary Data Table S5). Taking root EC as a measure of root system size, the estimate of root: shoot ratio (equal to root EC: shoot biomass) within semi-dwarf genotypes was higher than for normal-type hybrids at zero N fertilization as well as at high N fertilization. A similar trend in root: shoot ratio was detected in the *in vitro* test of near-isogenic hybrids (Table 5).

## DISCUSSION

The effect of the *bzh* gene was studied in plant material with a broad genetic background, because DH test hybrids resulted from a cross between a resynthesized line and a variety and the eight pairs of near-isogenic test hybrids were obtained from six mother and four father parental lines (Supplementary Data Table S1). ‘Shovelomics’ in near-isogenic hybrids at the end of vernalization (February 2017) in the field under high N fertilization, when applied as described by Trachsel *et al.* (2011) in maize, revealed no significant effect of *bzh* on root fresh matter, tap root length or basal stem diameter, but *bzh* semi-dwarf genotypes had a significantly shorter hypocotyl (data not shown). *bzh* semi-dwarfs have reduced shoot growth due to action of the *rga* gene. Stunted shoot growth can be observed in the field as early as autumn (Hofmann and Christen, 2007) and throughout the season (Foisset *et al.*, 1995; Miersch *et al.*, 2016*a*). Moreover, a reduced hypocotyl length of near-isogenic hybrids is observed early after germination under *in vitro* growth conditions (Table 5). The reduction in *in vitro* root growth was relatively small compared to that for hypocotyl, resulting in a higher root: shoot ratio in *bzh* semi dwarfs compared to normal-type near-isogenic hybrids.

### Nitrogen level effects on root system size

The overall increase in root EC under high N compared to zero N fertilization (Table 2) is in accordance with findings of Rudloff (2016), who analysed root EC in five environments in a set of 29 highly diverse winter oilseed rape genotypes and detected a mean of 2.36 nF at zero N and 3.73 nF at high N supply at the beginning of flowering and 1.89 and 2.63 nF, respectively, at maturity. Assuming a positive relationship between root biomass and root EC as assumed by Wu *et al.* (2016) in rapeseed, root growth was increased at high N in all field trials. This root growth reaction to N fertilization was also found in studies in controlled environments, such as by Svecnjak and Rengel (2006), Stahl (2015) and He *et al.* (2017) in glasshouse pot experiments.

The *bzh* semi-dwarf and normal-type genotypes responded differently to N fertilization. While semi-dwarfs outperformed normal-type genotypes in root EC at high N supply, this trend was inversed at low N fertilization, where semi-dwarfs had a reduced root EC in most trials. One exception to this trend was found in the late-season root EC measurement of DH test hybrids on 27 June, 2011 (Table 2), when normal-type genotypes had a significantly higher root EC at both N levels. In the 2010–11 season, N_min_ was low, at 27 kg N ha^−1^ at the beginning of plant growth, and the spring was dry (Table 1), with the first relevant summer rainfall at the beginning of June (6 June, 2011, 19.5 mm), long after flowering and towards the end of the pod-filling period. These climate conditions resulted in less N fertilizer being transported to the root zone and, as a consequence, a lower than expected N supply was present in the fertilized plots. Both N-level variants could therefore be considered as low N-fertilized, explaining the similar reaction of semi-dwarfs and normal types at both N levels.

Earlier studies on N use efficiency in DH test hybrids (Miersch *et al.*, 2016*b*) described QTL in the genomic region of the *bzh*-locus on linkage group A06 with a positive effect of the semi-dwarf *bzh* allele on five N efficiency-related parameters at zero N fertilization (N in seeds, N harvest index, N uptake efficiency, N utilization efficiency, N use efficiency). In the study of Miersch *et al.* (2016*b*), no QTL was detected in the genomic region of the single environment root EC QTL (Table 4) at linkage groups A03 and C08. QTL for root parameters in normal-type oilseed rape were estimated by Wang *et al.* (2017), who analysed QTL for root morphological traits in a rapeseed recombinant inbred line population under two contrasting N levels in hydroponics. They did not detect any QTL in the genomic regions of the QTL detected for root EC in this study. Neither did Bouchet *et al*. (2014, 2016), who estimated QTL for N use efficiency-related traits under medium and high N supply in a multi-environment field study, or Fletcher *et al.* (2015), who detected four QTL for root pulling force at two drought treatments in an oilseed rape DH population.

### Does tap root architecture of rapeseed influence root EC measurements?

Wu *et al.* (2016) calculated the cross-sectional area at the basal stem segment near the root crown of *B. napus* and assumed that stem diameter at the hypocotyl provided an indirect estimate of the cone-type form of the tap root volume. Correlations between root EC and stem diameter in our study were significant over all genotypes (Fig. 3) and, with *r* = 0.70* and 0.66* at the beginning and at the end of flowering, respectively, were lower than described by Wu *et al.* (2016; *r* = 0.87**). However, correlations within the groups of semi-dwarf genotypes and within the zero N or high N fertilization groups did not confirm this close relationship, showing smaller or negative correlations, such as *r* = −0.13 and 0.25 within high N at the beginning and the end of flowering, respectively. It can be concluded that root EC and stem diameter (as a proxy for tap root diameter) are not completely independent traits, although it is clear that stem diameter explains only part of the root EC values. As an example, if genotypes were selected based on root EC at zero N and within semi-dwarfs (Fig. 3B), the selection based on stem diameter would have excluded the genotype with the highest root EC. Because root parameters had not been determined in the field, an explanation for this correlation would be speculative (example.g. the influence of lateral and fine root systems). However, in most of our trials EC had a relatively high heritability, indicating that this method is suitable also in rapeseed to measure genetic variation in root system size.

### Root EC and nitrogen efficiency

Genotypes with high N efficiency need less N-fertilizer per unit yield, which increases the economic gain of the producer and prevents N leaching to the environment. The *bzh* semi-dwarf hybrids have higher N uptake, N utilization and N use efficiency than normal types at low N fertilization (Miersch *et al*., 2016*b*; Supplementary Data Table S4). In previous studies it was assumed that a reduction in shoot size and the resulting higher harvest index can explain the increase in N efficiencies by a more economic distribution of assimilates and nutrients, because shoot biomass is reduced but seed yield remains equal (Nyikako *et al.*, 2014; Stahl, 2015; Miersch *et al.*, 2016*b*). Additional to changes in harvest index, the increase in N efficiencies may be explained if *bzh* semi-dwarfs are not only altered in shoot size and architecture but also in root size and morphology. Differences in the *B. napus* root system were discussed earlier as an explanation for variation in N efficiency. Stahl (2015) found significant variation in the root: shoot ratio when testing a diverse set of *B. napus* genotypes at different N levels. Svecnjak and Rengel (2006) analysed four canola cultivars and detected a higher root: shoot ratio in N-efficient genotypes. In normal-type oilseed rape, a higher root: shoot ratio can be achieved by larger root systems, but this requires a higher investment of plant assimilates and energy. In *bzh* semi-dwarf hybrids, in which the shoot is reduced in size, a higher root: shoot ratio is acquired as soon as the *bzh* dwarfing gene reduces root growth relatively less than shoot growth.

This effect was found at zero N in this study, where root EC in *bzh* semi-dwarf hybrids was 7 % lower than in normal types (Table 2, Göttingen 2011–12), whereas the reduction in shoot biomass of semi-dwarfs compared to normal-type hybrids was higher with 15 % (Miersch *et al.*, 2015; Supplementary Data Table S4). In accordance with the conclusion of Svecnjak and Rengel (2006), we suggest that *bzh* semi-dwarfs were not only more N efficient than normal types when N is scarce due to a higher harvest index but also due to a higher root: shoot ratio. The higher root biomass (root EC) of normal-types is probably needed under low N to maintain biomass and yield, whereas the semi-dwarf hybrids have less biomass to be maintained. At high N supply, shoot biomass was reduced by 9 % in semi-dwarf compared to normal-type hybrids (Miersch *et al.*, 2015; Supplementary Data Table S4), whereas root EC was increased by approx. 20 % (Table 2). Here, the higher root EC: shoot biomass ratio of semi-dwarf hybrids resulted not only from reduced shoot biomass, but also from increased root EC. Wojciechowski *et al.* (2009) analysed root dry matter in different dwarf genotypes of wheat at sufficient N supply, and detected an increase in root dry matter in *Rht* dwarf types analogously to the *bzh* semi-dwarf at sufficient N fertilization in early plant development in autumn (Hofmann and Christen, 2007) and throughout the season in this study (Table 2).

Regarding the relationship between N uptake efficiency at the end of flowering and root EC, a lower root EC in semi-dwarf test hybrids correlated with a higher N uptake efficiency at the end of flowering (Fig. 4B; Göttingen 2011–12). This trend was reversed in the group of normal-type test hybrids, in which higher root EC correlated with higher N uptake efficiency, which confirmed the results of Rudloff (2016; *r* = 0.52 and *r* = 0.23 and at low and high N fertilization, respectively).

We conclude that the opposite root growth reaction of the *bzh* semi-dwarf and the normal type under N deficiency (Fig. 3) contributes to the higher N efficiency of semi-dwarf genotypes due to better economic use of N and assimilates. However, the effect of the *bzh* gene on the root system is complex and could be explained by a greater fine root system, altered lateral roots, changes in the tap root biomass or other shifts in root architecture. Additionally, the molecular and biochemical effects of an altered gibberellic acid metabolism in the *bzh* dwarf mutant on N uptake, N transport, and N partitioning has not yet been elucidated. The initial question of whether root EC may explain the higher N efficiency of *bzh* semi-dwarfs cannot be finally answered based on the available data. Consequently, an analysis of the root architecture of semi-dwarf and normal-type hybrids, as well as an analysis of the molecular and physiological plant reactions, would be needed to fully explain differences in N efficiency between the growth types.

## CONCLUSION

Our results indicate that *bzh* semi-dwarf hybrids have a reduced root system at a very early stage of plant development, as observed in *in vitro* seedling growth assays. In field experiments, semi-dwarf hybrids had lower root EC values only under low N supply, but not under high N supply. A QTL for this phenomenon was identified at the position of the *bzh* gene. Stem diameter and root EC correlations varied in the four growth-type/N-level combinations, and therefore stem diameter cannot be recommended as a general estimate for root EC. Further experiments are required to understand the higher N efficiency of *bzh* semi-dwarf hybrids, which can partly be explained by a higher harvest index, but also by an adapted root growth as a reaction to high or low N supply.

## SUPPLEMENTARY DATA

Supplementary data are available online at https://academic.oup.com/aob and consist of the following. Table S1: Parental genotypes of near-isogenic hybrid pairs. Table S2: Components of variance and respective F tests of root electrical capacitance from the analysis of variance combined across nitrogen levels. Table S3: Components of variance and respective F tests of seedling root parameters in three experiments. Table S4: Mean values of nitrogen (N) efficiency-related traits at the end of flowering (EOF) and at maturity. In total, 54 semi-dwarf and 54 normal-type hybrids were tested at low and high N supply at two locations, Einbeck and Göttingen, in the seasons 2010–11 and 2011–12. Table S5: (A) Correlation coefficients of root electrical capacitance with biomass and nitrogen uptake efficiency at the end of flowering and at maturity; (B) root: shoot ratio estimate on the basis of root EC and shoot biomass.

## Supplementary Material

mcy197_suppl_Tables
