# Supplementary material for: Root system size response of bzh semi-dwarf oilseed rape hybrids to different nitrogen levels in the field
Source: Ann Bot. 2018 Nov 17;124(6):891–901. doi: 10.1093/aob/mcy197 (PMC6881224; doi:10.1093/aob/mcy197)
Supplement: mcy197_suppl_Tables [file mcy197_suppl_tables.docx]

**Supplementary Data Table S1:** Parental genotypes of near-isogenic hybrid pairs

| **Near-isogenic …** | **male sterile ♀ line** | **♂ pollen parent line** |
| --- | --- | --- |
|  |  |  |
| Hybrid pair 1 |  |  |
| Semi-dwarf hybrid 1 | ♀ dwarf A1 | ♂ normal-type B1 |
| Normal-type hybrid 1 | ♀ normal-type A1 | ♂ normal-type B1 |
|  |  |  |
| Hybrid pair 2 |  |  |
| Semi-dwarf hybrid 2 | ♀ dwarf A2 | ♂ normal-type B2 |
| Normal-type hybrid 2 | ♀ normal-type A2 | ♂ normal-type B2 |
|  |  |  |
| Hybrid pair 3 |  |  |
| Semi-dwarf hybrid 3 | ♀ dwarf A2 | ♂ normal-type B3 |
| Normal-type hybrid 3 | ♀ normal-type A2 | ♂ normal-type B3 |
|  |  |  |
| Hybrid pair 4 |  |  |
| Semi-dwarf hybrid 4 | ♀ dwarf A3 | ♂ normal-type B2 |
| Normal-type hybrid 4 | ♀ normal-type A3 | ♂ normal-type B2 |
|  |  |  |
| Hybrid pair 5 |  |  |
| Semi-dwarf hybrid 5 | ♀ dwarf A3 | ♂ normal-type B4 |
| Normal-type hybrid 5 | ♀ normal-type A3 | ♂ normal-type B4 |
|  |  |  |
| Hybrid pair 6 |  |  |
| Semi-dwarf hybrid 6 | ♀ dwarf A4 | ♂ normal-type B1 |
| Normal-type hybrid 6 | ♀ normal-type A4 | ♂ normal-type B1 |
|  |  |  |
| Hybrid pair 7 |  |  |
| Semi-dwarf hybrid 7 | ♀ dwarf A5 | ♂ normal-type B1 |
| Normal-type hybrid 7 | ♀ normal-type A5 | ♂ normal-type B1 |
|  |  |  |
| Hybrid pair 8 |  |  |
| Semi-dwarf hybrid 8 | ♀ dwarf A6 | ♂ normal-type B1 |
| Normal-type hybrid 8 | ♀ normal-type A6 | ♂ normal-type B1 |

**Supplementary Data Table S2:** Components of variance (Var.cp.) and respective *F* tests of root electrical capacitance from the analysis of variance combined across nitrogen levels. 108 DH test hybrids (54 semi-dwarf and 54 normal type) and 16 near-isogenic hybrids (8 semi-dwarf and 8 normal type) were tested. Negative estimates for variance components are given by '0'. Heritabilities were estimated within growth-type in DH test hybrids (*h²*_G:T_) and for 16 near-isogenic hybrids (*h²*_G_).

| **Source** | **DH test hybrids** | | | | | | | |  |  | |  | **Near-isogenic hybrids** | | | | |  | |
| --- | --- | --- | --- | --- | --- | --- | --- | --- | --- | --- | --- | --- | --- | --- | --- | --- | --- | --- | --- |
|  |  | | | | | | | |  |  | |  |  | | | | |  | |
|  | Göttingen  2010-11 | |  | Göttingen  2011-12 | |  | Einbeck  2011-12 | |  |  | |  | Göttingen  2016-17 | | | | |  |  |
|  |  |  |  |  |  |  |  |  |  |  |  |  |  | BBCH 61 |  | BBCH 69 | | |  |
|  | Df **^a^** | Var.cp |  | Df | Var.cp |  | Df | Var.cp |  |  |  |  | Df | Var.cp |  | Var.cp | | |  |
|  |  |  | | | | | | | | | | | | | | |  |  |  |
|  |  |  |  |  |  |  |  |  |  |  |  |  |  |  |  |  |  |  |  |
| Nitrogen (N) | 1 | 0.021+ |  | 1 | 0.323* |  | 1 | 0.545** |  |  | N |  | 1 | 4.235** |  | 0.293 |  |  |  |
| Replication (R) :N |  |  |  | 2 | 0.029* |  | 2 | 0.014 |  |  | R:N |  | 2 | 0.021 |  | 0.419 |  |  |  |
| Growth type (T) | 1 | 0.006** |  | 1 | 0.032** |  | 1 | 0.025+ |  |  | T |  | 1 | 0 |  | 0 |  |  |  |
| T × N | 1 | 0 |  | 1 | 0.185** |  | 1 | 0.109* |  |  | T × N |  | 1 | 0.215* |  | 0 |  |  |  |
| (T × R) : N |  |  |  | 2 | 0 |  | 2 | 0.008 |  |  | (T × R) : N |  | 2 | 0.002 |  | 0.098** |  |  |  |
|  |  |  |  |  |  |  |  |  |  |  | G |  | 7 | 0.052** |  | 0.125** |  |  |  |
| G : T | 106 | 0 |  | 106 | 0.013* |  | 22 | 0.011 |  |  | G × T |  | 7 | 0.019 |  | 0.155** |  |  |  |
| (G × N) : T | 104 | 0.056 |  | 106 | 0 |  | 22 | 0 |  |  | G × N |  | 7 | 0 |  | 0.045* |  |  |  |
| (G × T) : (B × N) |  |  |  | 212 | 0.145 |  | 44 | 0.116 |  |  | (G × T × W) : N |  | 42 | 0.081** |  | 0.021 |  |  |  |
|  |  |  |  |  |  |  |  |  |  |  | (G×T×W×N) : Plant |  | 736 | 1.269 |  | 1.485 |  |  |  |
|  |  |  |  |  |  |  |  |  |  |  |  |  |  |  |  |  |  |  |  |
| *h² _G_*_:_*_T_* |  | *-* |  |  | *0.27* |  |  | *0.27* |  |  | *h² _G_* |  |  | *0.36* |  | *0.73* |  |  |  |

**^a^** Df; degrees of freedom

+Significant at the 0.1 probability level

*Significant at the 0.05 probability level

**Significant at the 0.01 probability level

**Supplementary Data Table S3:** Components of variance (Var.cp.) and respective *F* tests of seedling root parameters in three experiments. Seven near-isogenic pairs of test hybrids were scored *in vitro* conditions after 7 days on Hoagland medium. Heritabilities were estimated for 14 near-isogenic hybrids (*h²*_G_). Negative estimates for variance components are given by '0'.

|  | | Main root length  (cm) | Sum of lateral root length  (cm) | Total root length  (cm) | Number of lateral roots | Hypocotyle length  (cm) | Root : Shoot  ratio |
| --- | --- | --- | --- | --- | --- | --- | --- |
| Source | Df **^a^** | Var.cp | Var.cp | Var.cp | Var.cp | Var.cp | Var.cp |
| Experiment (E) | 2 | 0.859* | 6.774** | 3.909** | 1.240^+^ | 0.0003 | 4.894** |
| Replication (R) : E | 4 | 0.379** | 0.131 | 0.829* | 0.680 | 0.0010 | 0.228 |
| Growth type (T) | 1 | 0.018^+^ | 0.089^+^ | 0.197* | 1.085* | 0.0174** | 3.394** |
| T × E | 2 | 0 | 0.070 | 0.019 | 0.181 | 0 | 0.226 |
| Genotype (G) | 6 | 0.439** | 0.330** | 1.056** | 0.674** | 0.0058** | 4.766** |
| G × T | 6 | 0.063^+^ | 0.128* | 0.147^+^ | 0.524* | 0.0057** | 0.535* |
| G × E | 12 | 0.440** | 0.634** | 1.039** | 1.659** | 0.0041** | 2.559** |
| G × T × E | 12 | 0.853** | 0.096 | 1.391** | 0.350 | 0.0010 | 2.917** |
| (G × T × R) : E | 78 | 0.002 | 0 | 0.090 | 0.452 | 0.0033** | 0.366 |
| Plant: (R × G × T × E) | 492 | 2.543 | 3.574 | 6.821 | 12.814 | 0.014 | 13.535 |
|  |  |  |  |  |  |  |  |
| *h² _G_* |  | 0.87 | 0.79 | 0.83 | 0.74 | 0.94 | 0.94 |

**^a^** Df; degrees of freedom

+Significant at the 0.1 probability level

*Significant at the 0.05 probability level

**Significant at the 0.01 probability level

**Supplementary Data Table S4**: Mean values of nitrogen (N) efficiency related traits at the end of flowering (EOF) and at maturity. 54 semi-dwarf and 54 normal type hybrids were tested at low and high N supply in two locations Einbeck and Göttingen in the seasons 2010-2011 and 2011-2012.

|  | **Environment ^a^**    **Trait** | **Göttingen**  **2010-11** | **Einbeck 2011-12** | **Göttingen 2011- 12** |  | **Einbeck 2011-12** | **Göttingen 2011- 12** |
| --- | --- | --- | --- | --- | --- | --- | --- |
|  |  | ***--------------------low nitrogen----------------*** | | |  | ***------high nitrogen------*** | |
|  | **Electrical capacitance (EOF)** | | | | | | |
|  | Semi-dwarf | 0.88 | [1.4]^a^ | 2.28 |  | [2.8] ^a^ | 3.53 |
|  | Normal type | 0.98* | [1.5]** | 2.45^+^ |  | [2.2]^ns^ | 2.84** |
|  |  | | | | | | |
| **Data from Miersch (2015)** | **Biomass EOF (ton ha^-1^)** | | | | | | |
|  | Semi-dwarf | 4.83 | 1.22 | 1.45 |  | 3.05 | 3.85 |
|  | Normal type | 5.93** | 1.71** | 1.71** |  | 4.50** | 4.24** |
|  | **N content biomass EOF (%)** | | | | | | |
|  | Semi-dwarf | 1.75 | 2.11 | 1.86 |  | 2.83 | 3.81 |
|  | Normal type | 1.53** | 1.77** | 1.60** |  | 2.38** | 3.35** |
|  | **N in biomass EOF (kg N ha^-1^)** | | | | | | |
|  | Semi-dwarf | 84.4 | 25.7 | 27.4 |  | 85.9 | 146.4 |
|  | Normal type | 90.6* | 30.1** | 27.3 ^ns^ |  | 106.7** | 141.3** |
|  | **N uptake efficiency** ^b^ **EOF** | | | | | | |
|  | Semi-dwarf | 0.58 | 0.53 | 0.65 |  | 0.38 | 0.67 |
|  | Normal type | 0.62* | 0.63** | 0.65 ^ns^ |  | 0.47** | 0.64** |
|  |  | | | | | | |
| **Data from Miersch et al. (2016b)** | **N uptake efficiency** ^b^ **maturity** | | | | | | |
|  | Semi-dwarf | 0.76 | - | 0.83 |  | - | 0.55 |
|  | Normal type | 0.65** | - | 0.75** |  | - | 0.50** |
|  | **N utilization efficiency** ^c^ **maturity** | | | | | | |
|  | Semi-dwarf | 24.1 | - | 26.9 |  | - | 23.3 |
|  | Normal type | 21.3** | - | 24.2** |  | - | 22.4** |
|  | **N use efficiency** ^d^ **maturity** | | | | | | |
|  | Semi-dwarf | 18.3 | - | 22.4 |  | - | 12.7 |
|  | Normal type | 13.7** | - | 18.2** |  | - | 11.3** |
|  |  |  |  |  |  |  |  |
| **Data from Miersch et al. (2016a)** | **Seed yield Mg ha^-1^** | | | | | | |
|  | **Semi-dwarf** | **1.70*** | **-** | **1.28**** |  | **-** | **2.97**** |
|  | **Normal type** | **1.29** | **-** | **1.05** |  | **-** | **2.64** |
|  |  |  |  |  |  |  |  |

^a^ A reduced set of 12 genotypes was measured at maturity

**^b^ N uptake efficiency = total N uptake / total N supply (Moll et al. 1982)**

**^c^ N utilization efficiency = Seed yield / total N supply (Moll et al. 1982)**

**^d^ N use efficiency = Seed yield/ total N supply (Moll et al. 1982)**

**Supplementary Data Table S5**: **a)** Correlation coefficients of root electrical capacitance (EC) with biomass and nitrogen (N) uptake efficiency at the end of flowering (EOF) and at maturity and **b)** root:shoot ratio estimate on the basis of root EC and shoot biomass (EOF). 54 semi-dwarf and 54 normal type hybrids were tested at low and high N supply in Göttingen (2011-12). N efficiency data from Miersch (2015) and Miersch *et al.* 2016b

| a) | Root EC (EOF) | | | | |
| --- | --- | --- | --- | --- | --- |
|  |  | **N0** |  |  | **N1** |
|  | Semi-dwarf | Normal type |  | Semi-dwarf | Normal type |
|  |  |  |  |  |  |
| Biomass EOF (ton ha^-1^) | -0.27** | 0.31* |  | -0.19 | 0.37** |
| N uptake efficiency EOF | -0.31* | 0.25 |  | -0.11 | 0.18 |
|  |  |  |  |  |  |
| N use efficiency maturity | 0.00 | -0.11 |  | 0.00 | 0.15 |
| N uptake efficiency maturity | -0.04 | -0.04 |  | 0.03 | 0.12 |
| N use efficiency maturity | 0.07 | -0.17 |  | -0.06 | 0.05 |
| b)  Estimate of |  |  |  |  |  |
| Root : shoot ratio EOF | 1.59 | 1.44 |  | 0.92 | 0.67 |

*Significant at the 0.05 probability level

**Significant at the 0.01 probability level
